# Stimulation of DC-CIK with PADI4 Protein Can Significantly Elevate the Therapeutic Efficiency in Esophageal Cancer

**DOI:** 10.1155/2019/6587570

**Published:** 2019-03-03

**Authors:** Chunyan Liu, Yingying Zheng, Junyi Tang, Dawei Wang, Zhenshen Ma, Shutong Li, Xiaotian Chang

**Affiliations:** ^1^Medical Research Center of Shandong Provincial Qianfoshan Hospital, Shandong University, Jingshi Road 16766, Jinan, Shandong 250014, China; ^2^Medical Imaging Department of Shandong Provincial Qianfoshan Hospital, Shandong University, Jingshi Road 16766, Jinan, Shandong 250014, China; ^3^Shandong Clinical Medicine Research Center for Rheumatical Disease, Jingshi Road 16766, Jinan, Shandong 250014, China; ^4^Shandong Provincial Laboratory for Rheumatical Disease and Translational Medicines, Jingshi Road 16766, Jinan, Shandong 250014, China

## Abstract

**Background:**

PADI4 has extensive expression in many tumors. This study applied PADI4 as a tumor marker to stimulate DC- (dendritic cell-) CIK (cytokine-induced killer), an immunotherapy approach.

**Methods:**

A PADI4 expression plasmid was transfected into EC-originating ECA-109 cells. PADI4 gene was also inserted into a prokaryotic expression vector to produce recombinant protein. Lysate from PADI4-overexpressing cells or the purified recombinant PADI4 protein was used to load DCs, and the cells were then coincubated with CIK cells. DC and CIK cell phenotypes were determined using flow cytometry. The proliferation and viability of CIK cells were analyzed using trypan blue staining. The cytotoxic effect of DC-CIK cells on cultured ECA-109 cells was determined using CCK8 assays. Tumor-bearing mice were prepared by injection of ECA-109 cells. DC-CIK cells stimulated with lysate from PADI4-overexpressing cells or the PADI4 recombinant protein were injected into the tumor-bearing mice. The tumor growth was measured with magnetic resonance imaging (MRI).

**Results:**

Following incubation with lysate from PADI4-overexpressing cells, the ratio of CD40^+^ DCs increased by 17.5%. Induction of CIK cells with PADI4-stimulated DCs elevated the cell proliferation by 53.2% and the ability of CIK cells to kill ECA-109 cells by 12.1%. DC-CIK cells stimulated with lysate from PADI4-overexpressing cells suppressed tumor volume by 18.6% in the tumor-bearing mice. The recombinant PADI4 protein showed a similar effect on CIK cell proliferation and cytotoxicity as that of the lysate from PADI4-overexpressing cells. Furthermore, the recombinant protein elevated the ratio of CD40^+^ DCs by 111.8%, CD80^+^ DCs by 6.3%, CD83^+^ DCs by 30.8%, and CD86^+^ DCs by 7.8%. Induction of CIK cells with rPADI4-stimulated DCs elevated the cell proliferation by 50.3% and the ability of CIK cells to kill ECA-109 cells by 14.7% and suppressed tumor volume by 35.1% in the animal model.

**Conclusion:**

This study demonstrates that stimulation of DC-CIK cells with PADI4 significantly suppressed tumor growth in tumor-bearing mice by promoting DC maturation, CIK cell proliferation, and cytotoxicity. PADI4 may be a potential tumor marker that could be used to improve the therapeutic efficiency of DC-CIK cells.

## 1. Background

Dendritic cells (DCs) are the most potent antigen-presenting cells in the body [1]. Cytokine-induced killer (CIK) cells are a group of heterogeneous cells with CD3 and CD56 markers that possess the powerful antitumor activity of T cells and the non-MHC-restricted tumor-killing activity of natural killer cells [2]. DCs and CIK cells, as the major types of cells used in immunotherapy, can enhance the immune response and kill tumor cells via their cytotoxic activity [3–5]. The innate antigen-presenting capacity of DCs can effectively counteract the specificity deficiency of CIK cells and enhance their cytotoxicity [3]. Thus, the coculture of DCs with CIK cells (DC-CIK cells) has been used as a therapeutic strategy to treat malignant carcinomas such as esophageal cancer, non-small-cell lung cancer, and colorectal cancer [6–14].

Cultured tumor cells and tumor tissue lysates are common antigens used to load DCs in clinical immunotherapy. Pulsing DCs with these tumor-associated antigens can elevate their specific antitumor activity. However, the concentration of tumor-specific proteins in tumor tissues is often too low to sufficiently induce a potent immune response. Thus, a tumor marker with increased expression in tumor tissues and with wide distribution in various tumor types would be ideal for stimulating DCs to achieve high efficiency and to manufacture DC-CIK cell-based vaccines to treat various tumors.

Protein arginine deiminases isoform 4 (PADI4) participates in posttranslational protein modification by catalyzing the conversion of arginine to citrulline. Our studies and others have demonstrated that PADI4 expression is increased in many human tumor tissues, including cervical squamous cell carcinoma, gastric carcinoma, lung cancer, ovarian serous papillary adenocarcinoma, thyroid papillary carcinoma, and esophageal cancer [15–18]. An anticancer peptidylarginine deiminase (PAD) inhibitor is being considered as a potential drug to treat tumors [19]. Therefore, PADI4 may be a potential therapeutic target in tumors [20].

In the present work, we investigated the potential value of PADI4 as a tumor marker in DC-CIK cell immunotherapy. We used cultured esophageal cancer (EC) cells with overexpression of PADI4 or used a recombinant PADI4 protein as a tumor antigen to load DCs in DC-CIK cell immunotherapy of mouse-burden EC. We had demonstrated that PADI4 expression was increased in esophageal squamous cell carcinoma [18].

## 2. Methods

### 2.1. Ethics and Consent

Peripheral blood was donated by young, healthy volunteer donors (25-35 years old, *n* = 12, female = 6). The study was conducted in accordance with the Declaration of Helsinki and was approved by the Ethics Committee of Shandong Province Qianfoshan Hospital (approval number 2015004), Jinan, China. We obtained informed consent from all volunteers involved in the study. The mice were housed in an Association for Assessment and Accreditation of Laboratory Animal Care- (AAALAC-) accredited facility.

### 2.2. Overexpression of PADI4 in ECA-109 Cells

Total RNA was extracted from EC tissues using TRIzol reagent (Beyotime Biotechnology, Shanghai, China). The extracted RNA was reverse-transcribed into first-strand cDNA. The full cDNA sequence of PADI4 was amplified via PCR using the following specific primer pair: PADI4-EcoRI-Fex: TACTCAGAATTCATGGCCCAGGGGACATTG and PADI4-AscI-Rex: TACTCAGGCGCGCCGAGGGCACCATGTCCA. The PCR products were inserted into pcDNA3.1-RFP (red fluorescent protein). RFP was inserted into pcDNA3.1 as a reporter gene. The recombinant overexpression plasmid pcDNA3.1-PADI4-RFP was transfected into ECA-109 cells using PolyJet™ DNA in Vitro Transfection Reagent (SignaGen, Maryland, USA) according to the instruction of the manufacturer.

TXNDC5 was used as the nonspecific control in this study. The full cDNA sequence of TXNDC5 was amplified via PCR using a specific pair of primers: TXNDC5-BamHI-Fex: TACTCAGGATCCCATGGAAGATGCCAAA and TXNDC5-AscI-Rex: TACTCAGGCGCGCCGAAAGTTCGTCTTTCGC.

### 2.3. Preparation of ECA-109 Cell Lysates

Following successful transfection of PADI4, TXNDC5, or RFP recombinant plasmids into ECA-109 cells, the cells were centrifuged for 5 min at 1500 rpm and resuspended in 1 ml of sterile phosphate-buffered saline (PBS). The cell suspension was lysed via five freeze-thaw cycles (-80°C/37°C). The lysate was centrifuged at 12000 rpm for 15 min and filter sterilized. The supernatant was collected, and the protein concentration was determined using a Bradford Protein Assay Kit (Beyotime, China).

### 2.4. Prokaryotic Expression of the Recombinant PADI4 Protein

Total RNA was extracted from EC tissues using TRIzol reagent. The extracted RNA was reverse-transcribed into first-strand cDNA. Then, the cDNA was used as a template to amplify the full-length PADI4 sequence via PCR using the following specific primer pair: PADI4-EcoRI-Fex: TACTCAGAATTCATGGCCCAGGGGAC ATTG and PADI4-SalI-Rex: TACTCAGTCGACTCAGGGCACCATGTTCCA. The PCR products were sequenced and inserted into the pGEx-4T1 expression vector. pGEx-4T1 is a commonly used prokaryotic expression vector with ampicillin resistance and a GST tag for recombinant protein purification. The recombinant plasmid was transformed into competent *Escherichia coli* BL21 host cells. Expression of recombinant PADI4 was induced using isopropyl-*β*-D-thiogalactoside (0.1 mM) at 16°C overnight. The PADI4 recombinant protein (rPADI4) was expressed in a soluble form and purified using Glutathione Sepharose beads.

The recombinant PADI4 protein was digested using protease K (working concentration: 50 *μ*g/ml) at 58°C for 2 h as a control. The digested recombinant PADI4 protein (d-rPADI4) was then heated at 95°C for 1 h to make the enzyme inactive. r-PADI4 and d-rPADI4 were verified by SDS-PAGE with Coomassie brilliant blue staining.

### 2.5. Preparation of Peripheral Blood Mononuclear Cells (PBMCs)

Whole blood was centrifuged for 20 min at 2000 rpm. The pelleted cells were resuspended in PBS at a ratio of 1 : 1 and were separated by Ficoll-Hypaque density gradient centrifugation (Solarbio, Beijing, China) for 30 min at 2000 rpm. The cells in the white layer were collected, resuspended in 50 ml of PBS, and centrifuged for 15 min at 1500 rpm. The pelleted cells were resuspended in 50 ml of PBS and centrifuged for 15 min at 1300 rpm. The PBMC pellet was obtained after discarding the supernatant. The mononuclear cells were resuspended in 50 ml of PBS, counted using a hemocytometer, and examined with trypan blue dye solution.

### 2.6. Preparation of Monocyte-Derived DCs (MDDCs)

Monocytes were purified from the PBMCs using CD14 microbeads (Miltenyi, Germany). The purity of the monocytes was verified by flow cytometry (FCM). To induce monocytes to differentiate into DCs, the purified CD14^+^ monocytes were cultured in a humidified atmosphere of 5% CO_2_ at 37°C in RPMI-1640 culture medium supplemented with 1% penicillin/streptomycin, 10% FBS (Gibco, Life Technologies Corporation, Australia), 1 mM glutamine, 1000 U/ml human rGM-CSF (PeproTech, USA), and 1000 U/ml human rIL-4 (PeproTech, USA). Half of the medium was replaced every 2 days, and the MDDCs were collected after 6 days of culture. The purity of the MDDCs was >99% as determined by examining the expression of CD11c with FCM.

### 2.7. Promoting DC Maturation

After 6 days of culture in a humidified atmosphere of 5% CO_2_ at 37°C in RPMI-1640 culture medium (supplemented with 1% penicillin/streptomycin, 10% FBS, 1 mM glutamine, 1000 U/ml human rGM-CSF, and 1000 U/ml human rIL-4), 20 *μ*g/ml lysate from PADI4-overexpressing ECA-109 cells or rPADI4 was added to the MDDC culture medium, and the cells were incubated at 37°C with 5% CO_2_ for another 24 h to promote DC maturation. Lysate from TXNDC5- or RFP-overexpressing ECA-109 cells was used as a control for DC induction with lysate from PADI4-overexpressing cells, while digested rPADI4 (d-rPADI4) was used as a control for DC induction with rPADI4. After stimulation, the MDDCs were harvested. The expressions of cell surface molecules, including CD11c, CD40, CD80, CD83, and CD86, were measured by FCM using CD11c-PE-cy5, CD40-APC, CD80-PE-cy7, CD83-FITC, and CD86-PE FCM antibodies, respectively.

### 2.8. Preparation of CIK Cells

PBMCs were cultured in a humidified atmosphere of 5% CO_2_ at 37°C in RPMI-1640 culture medium supplemented with 1% penicillin/streptomycin and 10% FBS (Gibco, Life Technologies Corporation, Australia). On the first day of culture, 1000 U/ml interferon-*γ* (IFN-*γ*; BioLegend, USA) was added to the culture medium. After 24 h of culture, 50 ng/ml anti-CD3 antibody, 300 U/ml IL-2, and 100 U/ml IL-1*α* were added to the culture medium. Fresh culture medium including 1% penicillin/streptomycin, 10% FBS, 1 mM glutamine, and 300 U/ml IL-2 was added to the cells every two days at a ratio of 1 : 1.

### 2.9. Flow Cytometry

MDDCs were centrifuged at 1100 rpm for 10 min. After discarding the supernatant, the MDDCs were washed and resuspended in MACS buffer (0.5% FBS and 2 mM EDTA in PBS) and incubated with a PE-cy5-conjugated antibody against CD11c, an APC-conjugated antibody against CD40, a PE-cy7-conjugated antibody against CD80, a FITC-conjugated antibody against CD83, or a PE-conjugated antibody against CD86 at room temperature for 30 min in the dark place. After washing with MACS buffer, the MDDCs were fixed with 200 *μ*L of 1% paraformaldehyde and analyzed by FCM.

### 2.10. Cell Proliferation and Activity Analysis Using Trypan Blue Staining

Cells were centrifuged at 1100 rpm for 10 min. After discarding the supernatant, the cells were resuspended in PBS. Then, 90 *μ*l of the cell suspension was mixed with 10 *μ*l of trypan blue staining buffer (0.4%), and the number of cells was counted using a microscope. Dead cells were stained blue, while living cells were colorless.

### 2.11. Coculture of MDDCs with CIK Cells

rPADI4 or lysate from PADI4-overexpressing ECA-109 cells was used as an antigen to load MDDCs at the concentration of 20 *μ*g/10^6^ cells. The MDDCs were cultured in a humidified atmosphere of 5% CO_2_ at 37°C in RPMI-1640 culture medium supplemented with 1% penicillin/streptomycin (Sigma-Aldrich, Shanghai, China) and 10% FBS (Gibco, Life Technologies Corporation, Australia). After 24 h of incubation, TNF-*α* (Peprotech, Rocky Hill, USA) (500 U/ml) was added to the culture medium, and the cells were incubated for another 24 h. The MDDCs were collected and cocultured with CIK cells at a ratio of 1 : 10; approximately 10^5^ MDDC cells were cultured with 10^6^ CIK cells per ml in RPMI-1640 culture medium supplemented with 300 U/ml IL-2 (Shandong Port of Spring Pharmaceutical, Jinan, China), 1% penicillin/streptomycin, and 10% FBS in a humidified atmosphere of 5% CO_2_ at 37°C for 6 days. Complete culture medium including 1% penicillin/streptomycin, 10% FBS, 1 mM glutamine (Sigma-Aldrich, Shanghai, China), and 300 U/ml IL-2 was added to the cells every two days. After 6 days of culture, the cocultured cells were harvested for further analysis.

### 2.12. In Vitro T Cell Cytotoxicity Assay

ECA-109 cells were seeded in a 96-well plate (5 × 10^3^ cells/well) in a humidified atmosphere of 5% CO_2_ at 37°C. After 24 h of culture, the culture medium was discarded, and 100 *μ*l of cocultured MDDCs + CIK cells (5 × 10^5^ cells/ml) was added to the cultured ECA-109 cells. The ECA-109 cells were adherent cells, and the MDDCs + CIK cells were suspension cells. After another 24 h of culture, the supernatant including MDDCs + CIK cells was discarded, and the surviving ECA-109 cells were left in the wells. After washed with PBS for three times, 100 *μ*l of Cell Counting Kit-8 (CCK8) solution mixture (Dojindo, Japan) (90 *μ*l RPMI-1640 complete culture medium + 10 *μ*l CCK8 solution) was added to each well, and the cells were incubated for 2 h. The absorbance at 450 nm was measured using a microplate reader. The cytotoxic effect of DC-induced CIK cells on ECA-109 cells was analyzed using the following formula: cell cytotoxicity = (1 − (*A*_e_ − *A*_b_)/(*A*_c_ − *A*_b_)) × 100%. In this formula, *A*_e_ is the absorbance of the experimental group, *A*_c_ is the absorbance of the control group, and *A*_b_ is the absorbance of a blank well.

### 2.13. Treating Tumor-Bearing Mice with PADI4-Stimulated DC-CIK Cells

BALB/c (Vital River, Beijing, China) nude mice at 5 weeks old were used to prepare the tumor-bearing animal model. Twenty BALB/c nude mice were injected with 200 *μ*l of ECA-109 cell suspension (10^7^ cells/ml in PBS) to generate tumor-bearing nude mice. These nude mice were randomly divided into two groups. Next, 20 *μ*g of lysate from PADI4-overexpressing ECA-109 cells was used to load DCs. The stimulated DCs were cocultured with CIK cells, and 200 *μ*l of DC-CIK cells was injected into surrounding tumor tissues of the tumor-bearing nude mice at 28 d, 29 d, 30 d, and 31 d (10^7^ cells/ml in PBS) after the ECA-109 cell injection. As a control, 20 *μ*g of lysate from RFP-overexpressing ECA-109 cells was used to load DCs, the DCs were cocultured with CIK cells, and 200 *μ*l of DC-CIK cells was injected into surrounding tumor tissues of the tumor-bearing nude mice at 28 d, 29 d, 30 d, and 31 d (10^7^ cells/ml in PBS) after the ECA-109 cell injection. Gross tumor volumes were measured at 28 d, 32 d, 36 d, 40 d, and 44 d. The mice were sacrificed on the 44th day after the tumor cell injection. The shape and volume of the tumors were examined using magnetic resonance imaging (MRI) before sacrifice.

By the same protocol, 20 *μ*g of recombinant PADI4 (rPADI4) protein was used to load DCs. The stimulated DCs were cocultured with CIK cells, and 200 *μ*l of DC-CIK cells was injected into surrounding tumor tissues of the tumor-bearing nude mice at 28 d, 29 d, 30 d, and 31 d (10^7^ cells/ml in PBS) after the ECA-109 cell injection. As a control, 20 *μ*g of d-rPADI4 was used to load DCs. The stimulated DCs were cocultured with CIK cells, and 200 *μ*l of DC-CIK cells was injected into surrounding tumor tissues of the tumor-bearing nude mice at 28 d, 29 d, 30 d, and 31 d (10^7^ cells/ml in PBS) after the ECA-109 cell injection.

### 2.14. Statistical Analysis

Data were analyzed by a two-tailed Student *t*-test. Differences were considered to be statistically significant at *p* < 0.05. To verify the results, each experiment was performed with three samples in triplicate.

## 3. Results

### 3.1. PADI4-Overexpressing ECA-109 Cell Lysates Can Elevate the Percentage of CD40^+^ DCs

Overexpression of PADI4 was induced by transfecting plasmids into ECA-109 cells. PADI4 showed significantly increased transcription and translation in ECA-109 cells transfected with the PADI4 expression plasmid compared with cells transfected with the RFP expression plasmid. TXNDC5 showed increased expression in ECA-109 cells transfected with a TXNDC5 expression plasmid, compared with cells transfected with the RFP expression plasmid. The result is shown in Supplementary Figure S1.

**Figure 1:**
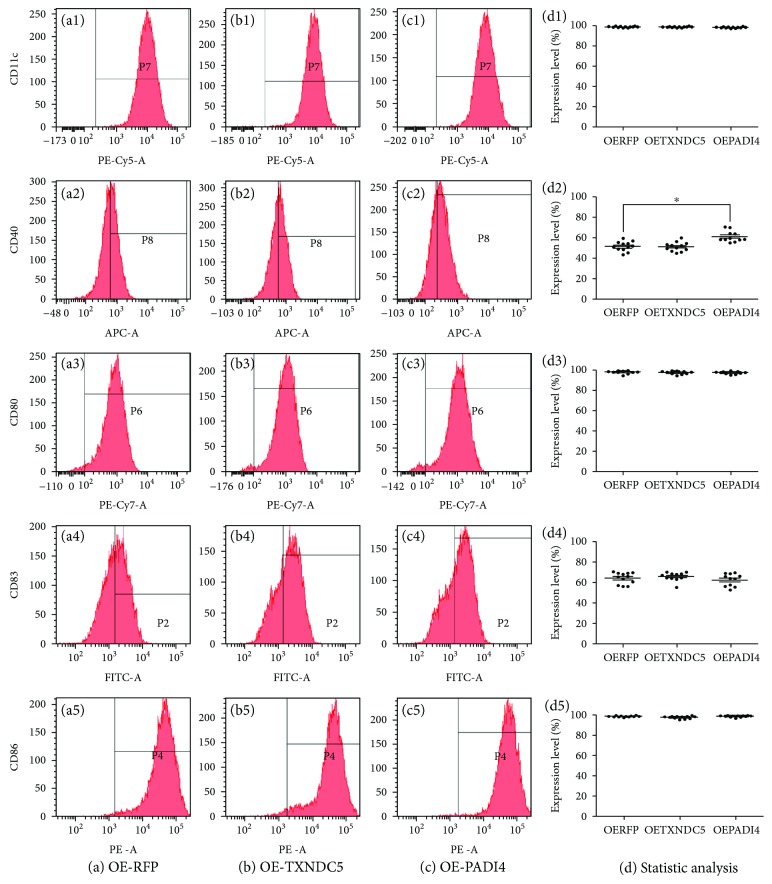
Detection of CD11c, CD40, CD80, CD83, and CD86 expressions in DCs using flow cytometry. DCs were incubated with lysates from ECA-109 cells transfected with an RFP expression plasmid (OE-RFP) (a), a TXNDC5 expression plasmid (OE-TXNDC5) (b), or a PADI4 expression plasmid (OE-PADI4) (c). Statistical analysis of the above flow cytometry results (d).

DCs were treated with lysate from PADI4-overexpressing ECA-109 cells. The percentage of CD11c^+^ DCs loaded with lysate from RFP-, TXNDC5-, or PADI4-overexpressing cells was 99.9%, 99.9%, and 99.9%, respectively, indicating the high purity of DCs in all three groups. The percentage of CD40^+^ DCs in the three groups was 51.9%, 50.1%, and 61.0%, respectively, indicating that PADI4 overexpression in ECA-109 cell lysates can elevate the percentage of CD40^+^ DCs. The percentage of CD80^+^ DCs in the three groups was 98.1%, 98.4%, and 97.9%, respectively. The percentage of CD83^+^ DCs in the three groups was 64.2%, 66.8%, and 63.0%, respectively. The percentage of CD86^+^ DCs in the three groups was 99.2%, 98.3%, and 99.0%, respectively. These measurements indicate that PADI4 did not significantly affect the percentages of CD80^+^, CD83^+^, and CD86^+^ DCs. The above results are shown in Figure 1.

### 3.2. PADI4-Overexpressing ECA-109 Cell Lysates Can Elevate DC-Induced CIK Cell Proliferation and Cytotoxicity

PADI4-overexpressing ECA-109 cell lysate-loaded DCs were cocultured with CIK cells as the experiment group. RFP-overexpressing ECA-109 cell lysate-loaded DCs were cocultured with CIK cells as the negative control group, and TXNDC5-overexpressing ECA-109 cell lysate-loaded DCs were cocultured with CIK cells as the nonspecific control group. Results showed that CIK cells cocultured with DCs loaded with lysate from PADI4-overexpressing ECA-109 cells, the ratio of CD3^+^CD4^+^ CIK cells was 14.3%, while that of CD3^+^CD8^+^ CIK cells was 82.8%, and that of CD3^+^CD16^+^CD56^+^ CIK cells was 39.4%. When CIK cells were cocultured with DCs loaded with lysate from TXNDC5-overexpressing ECA-109 cells, the ratio of CD3^+^CD4^+^ CIK cells was 13.8%, while that of CD3^+^CD8^+^ CIK cells was 82.3%, and that of CD3^+^CD16^+^CD56^+^ CIK cells was 38.5%. When CIK cells were cocultured with DCs loaded with lysate from RFP-overexpressing ECA-109 cells, the ratio of CD3^+^CD4^+^ CIK cells was 12.2%, while that of CD3^+^CD8^+^ CIK cells was 84.1%, and that of CD3^+^CD16^+^CD56^+^ CIK cells was 39.1%. These results indicate that lysate from PADI4-overexpressing ECA-109 cells did not significantly alter the phenotypic profile of CIK cells compared with the two control lysates. These results are shown in Supplementary Figure S2.

**Figure 2:**
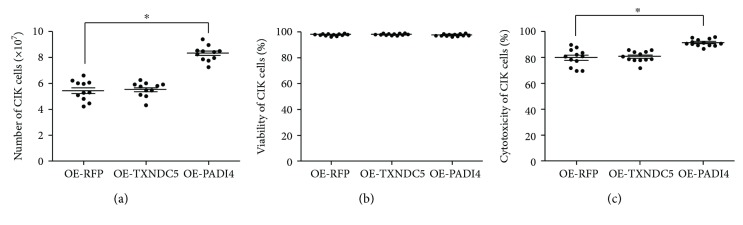
Detection of CIK cell proliferation, viability, and cytotoxicity. (a) CIK cells were induced with DCs loaded with lysate from RFP-overexpressing (OE-RFP), TXNDC5-overexpressing (OE-TXNDC5), or PADI4-overexpressing (OE-PADI4) ECA-109 cells. The number of CIK cells was counted following trypan blue staining. (b) CIK cell viability was analyzed following trypan blue staining. (c) A CCK8 kit was used to determine the number of ECA-109 cells following treatment with DC-CIK cells. Asterisks indicate significant differences between CIK cells cocultured with DCs loaded with lysate from PADI4-overexpressing cells and CIK cells cocultured with control DCs. ^∗^ indicates *p* < 0.05.

When CIK cells were cocultured with DCs loaded with lysate from PADI4-overexpressing ECA-109 cells, TXNDC5-overexpressing ECA-109 cells, or RFP-overexpressing ECA-109 cells, the CIK cell number was 8.06 × 10^7^, 5.44 × 10^7^, and 5.26 × 10^7^, respectively. These results indicate that PADI4 overexpression in ECA-109 cells significantly induced CIK cell proliferation compared with the two control groups. These results are shown in Figure 2(a). When CIK cells were cocultured with DCs loaded with lysate from PADI4-overexpressing ECA-109 cells, TXNDC5-overexpressing ECA-109 cells, or RFP-overexpressing ECA-109 cells, the CIK cell viability ratio was 97.5%, 97.9%, and 98.1%, respectively. These results indicate that PADI4 overexpression does not significantly affect cell viability compared with the two control groups. These results are shown in Figure 2(b). The above findings suggest that PADI4 overexpression in ECA-109 cells can promote DC-induced CIK cell proliferation but has no significant effect on the phenotype or viability of CIK cells. In addition, the cytotoxicity of the DC-CIK cells primed with lysate from PADI4-overexpressing cells, RFP-overexpressing cells, or TXNDC5-overexpressing cells was 90.7%, 80.9%, and 80.4%, respectively. These results indicate that overexpression of PADI4 in ECA-109 cells can significantly enhance the cytotoxic effect of DC-CIK cells on ECA-109 cells. These results are shown in Figure 2(c).

### 3.3. PADI4-Overexpressing ECA-109 Cell Lysates Can Enhance the Therapeutic Efficiency of DC-CIK Cells in Tumor-Bearing Mice

**Figure 3:**
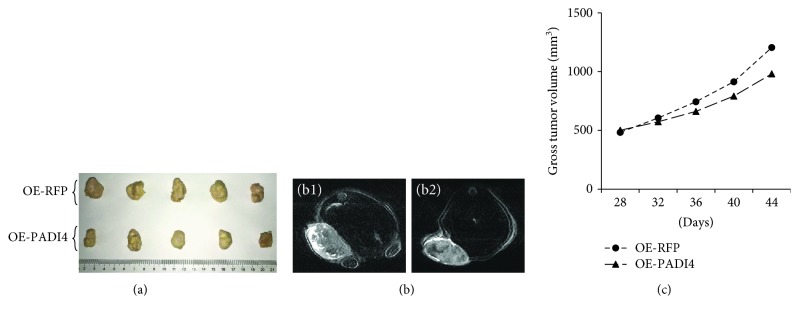
Detection of the therapeutic efficiency of DC-CIK cells in tumor-bearing mice. ECA-109 cells were injected into nude mice to generate tumor-bearing mice. At 28 d, 29 d, 30 d, and 31 d following the injection of the tumor cells, DC-CIK cells were injected into the tumor-bearing mice. DC-CIK cells induced with lysate from RFP-overexpressing or PADI4-overexpressing ECA-109 cells were injected into the mice. (a) Tumor tissues were dissected from the tumor-bearing mice at 44 d after the ECA-109 cell injection. (b) MRI was used to measure the shape and size of the tumors at 44 d after the tumor cell injection. (c) Gross tumor volume was analyzed at 28 d, 32 d, 36 d, 40 d, and 44 d after the tumor cell injection based on Vernier caliper measurements.

DC-CIK cells induced with lysate from PADI4-overexpressing ECA-109 cells were injected into nude mice bearing esophageal tumors. The average tumor volume in mice injected with PADI4-induced DC-CIK cells was 980 mm^3^, while the tumor volume in mice injected with RFP-stimulated DC-CIK cells was 1204 mm^3^. The result indicates that PADI4 overexpression in ECA-109 cells can enhance the treatment efficiency of DC-CIK cells in mice bearing tumors originating from ECA-109 cells. These results are shown in Figure 3.

### 3.4. Loading rPADI4 Increases the Ratios of CD40^+^, CD80^+^, CD83^+^, and CD86^+^ DCs

In this study, PADI4 gene was inserted into pGEx-4T1 plasmids and expressed in *Escherichia coli* BL21. The PADI4 protein was expressed in a soluble form and purified using Glutathione Sepharose beads. The recombinant PADI4 protein was digested using protease K as the control group. SDS-PAGE with Coomassie brilliant blue staining showed the successful digestion of the recombinant PADI4 protein (Supplementary Figure S3).

**Figure 4:**
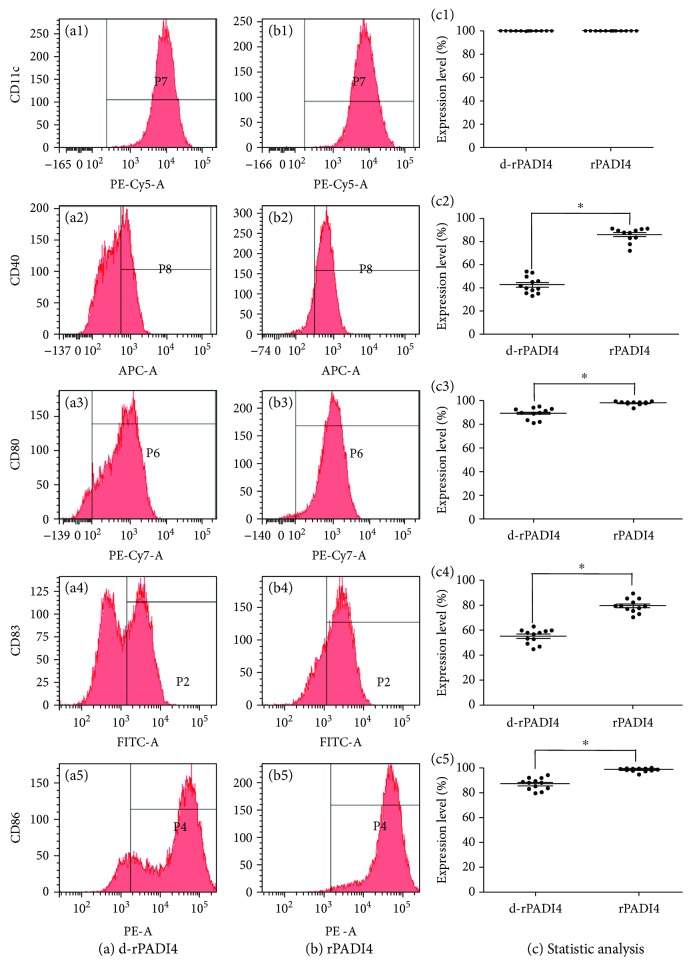
Detection of CD11c, CD40, CD80, CD83, and CD86 expressions on DCs using flow cytometry. DCs were loaded with (a) d-rPADI4 and (b) rPADI4. (c) Statistical analysis of the above FCM results. Asterisks indicate significant differences between DCs induced with rPADI4 and the control group. ^∗^ indicates *p* < 0.05.

In this study, rPADI4 was used to load DCs, while d-rPADI4 was used as a negative control. The percentage of CD11c^+^ DCs in both groups was 99.9%, indicating the high purity of these DCs. The ratio of CD40^+^ DCs in d-rPADI4-loaded DCs and rPADI4-loaded DCs was 41.5% and 87.9%, the ratio of CD80^+^ DCs was 92.7% and 98.5%, the ratio of CD83^+^ DCs was 57.7% and 75.5%, and the ratio of CD86^+^ DCs was 87.7% and 94.5%, respectively. These results indicate that rPADI4 can significantly increase the ratios of CD40^+^, CD80^+^, CD83^+^, and CD86^+^ DCs. These results are shown in Figure 4.

### 3.5. rPADI4-Loaded DCs Promote CIK Cell Proliferation and Enhance CIK Cytotoxicity

In this study, rPADI4 was used to load DCs, and the DCs were cocultured with CIK cells. Following induction of rPADI4-loaded DCs, the ratio of CD3^+^CD4^+^ CIK cells was 13.4%, while the ratio of CD3^+^CD8^+^ CIK cells was 82.5%, and the ratio of CD3^+^CD16^+^CD56^+^ CIK cells was 10.4%. In the CIK cells induced with d-rPADI4-loaded DCs, the ratios of CD3^+^CD4^+^, CD3^+^CD8^+^, and CD3^+^CD16^+^CD56^+^ CIK cells were 11.2%, 81.9%, and 11.8%, respectively. These results indicate that rPADI4 has no obvious effect on the phenotypic profile of CIK cells. These results are shown in Supplementary Figure S4.

**Figure 5:**
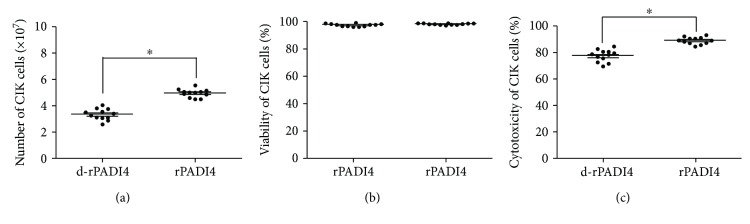
Detection of CIK cell proliferation, viability, and cytotoxicity. CIK cells were induced with DCs loaded with rPADI4. CIK cells induced with d-rPADI4-loaded DCs were used as a control. (a) The number of CIK cells was counted following coculture with DCs. (b) The viability of CIK cells was analyzed following coculture with DCs. (c) A CCK8 kit was used to determine the cytotoxic effect of DC-CIK cells on the cultured ECA-109 tumor cells. CIK cells induced with d-rPADI4-loaded DCs were used as a control. Asterisks indicate significant differences between the two groups. ^∗^ indicates *p* < 0.05.

When CIK cells were cocultured with d-rPADI4-loaded DCs or rPADI4-loaded DCs, the number of CIK cells was 3.22 × 10^7^ and 4.84 × 10^7^, respectively. These results indicate that rPADI4 can promote CIK cell proliferation. These results are shown in Figure 5(a). When CIK cells were cocultured with DCs loaded with d-rPADI4 or DCs loaded with rPADI4, the cell viability was 97.8% or 97.3%, respectively. These results suggest that rPADI4 can promote DC-induced CIK cell proliferation but has no significant effect on CIK cell viability. These results are shown in Figure 5(b). In addition, the cytotoxicity of DC-CIK cells induced with rPADI4 was 88.7%, while that of the CIK cells induced with d-rPADI4-loaded DCs was 77.3%. These results indicate that rPADI4 can significantly enhance the cytotoxic effect of DC-CIK cells on ECA-109 cells. These results are shown in Figure 5(c).

### 3.6. rPADI4-Loaded DCs Enhance the Therapeutic Efficiency of CIK in Tumor-Bearing Mice

**Figure 6:**
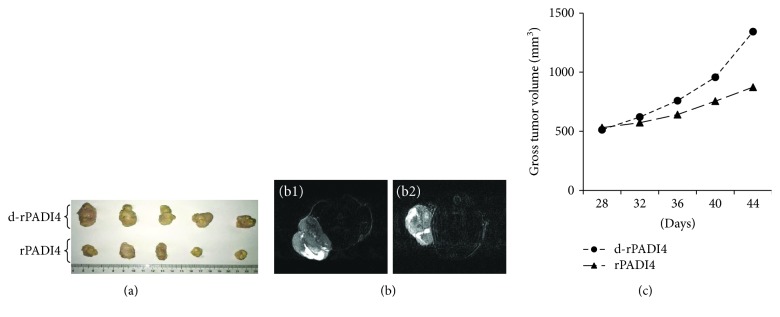
Measurement of the therapeutic efficiency of DC-CIK cells in tumor-bearing mice. ECA-109 cells were injected into nude mice to generate tumor-bearing mice. At 28 d, 29 d, 30 d, and 31 d after ECA-109 cell injection, DC-CIK cells were injected into the tumor-bearing mice. CIK cells were induced with DCs loaded with rPADI4. CIK cells induced with d-rPADI4-loaded DCs were used as control. (a) Tumor tissues were dissected from the tumor-bearing mice at 44 d after the ECA-109 cell injection. (b) MRI was used to measure the shape and size of the tumors at 44 d after the tumor cell injection. (c) Gross tumor volume was analyzed at 28 d, 32 d, 36 d, 40 d, and 44 d after the tumor cell injection based on Vernier caliper measurements.

CIK cells induced with rPADI4-loaded DCs were injected into nude mice bearing esophageal tumors. Following injection of d-rPADI4-induced DC-CIK cells, the average tumor volume in mice was 1343 mm^3^; following injection of rPADI4-induced DC-CIK cells, the average tumor volume in mice was 873 mm^3^. The result indicates that stimulation of DC-CIK cells with rPADI4 can significantly enhance treatment efficiency in mice bearing tumors. These results are shown in Figure 6.

## 4. Discussion

CD40 is a marker of DC maturation, enhances CD40 expression on the surface of dendritic cell can improve its antitumor immune response [21]. In this study, we found that PADI4-overexpressing ECA-109 cell lysate can promote the percentage of CD40^+^ DCs increased by 17.5% compared with control group. As well, rPADI4 elevated the ratio of CD40^+^ DCs by 111.8%, CD80^+^ DCs by 6.3%, CD83^+^ DCs by 30.8%, and CD86^+^ DCs by 7.8% compared with control group. These observations suggest that PADI4 can significantly stimulate DC maturation. There are some examples of interactions between DCs and self-molecules, especially the abnormal self-proteins such as many tumor antigens. MUC1, human epithelial cell antigen mucin, is recognized in its aberrantly glycosylated form on tumor cells. On encounter with MUC1, immature DCs increase cell surface expression of CD40, CD80, CD83, and CD86 molecules and the production of IL-6 and TNF-alpha cytokines [22]. Ovalbumin and monophosphoryl lipid A adjuvant were encapsulated within the nanoparticle and were cultured with DCs. Higher fold increase in surface activation markers such as CD40, CD86, and major histocompatibility complex class II molecules as well as its secretion of extracellular cytokines were significantly increased in this DCs [23]. To enhance DC-based vaccines, a synthetic ligand-inducible CD40 receptor in combination with lipopolysaccharide or monophosphoryl lipid A was used to culture DCs and consequentially led to high expression of DC maturation markers [23]. These studies indicate the maturation of DCs following the stimulation of self-proteins.

In the present study, after cocultured with DCs stimulated with lysate from PADI4-overexpressing ECA-109 cells or rPADI4, CIK cell proliferation was 53.2% or 50.3% higher compared with the control group. CIK cell cytotoxicity was elevated by 12.1% or 14.7% compared with the control group. These results demonstrate that loading DCs with PADI4 and using them induce CIK cells can enhance cell proliferation of the CIK and its cytotoxic effect on tumor cells. Many studies have reported that the proliferation, phenotype, and antitumor activity of CIK cells are significantly enhanced following coculture with DCs in vitro. For example, the cytotoxic effect of DC-CIK cells on HL-60 leukemia cells, HeLa cervical cancer cells, and HepG2 liver cancer cells was much stronger than that of CIK cells alone [24–29]. DC transfected by recombinant adeno-associated virus can stimulate the proliferation and differentiation of lymphocytes and also induce the proliferation of cytotoxic T lymphocytes, and their own phenotypes are not significantly altered [30]. CIK proliferation, differentiation, and cytotoxicity were enhanced by coculturing with the DCs that were pulsed with complete tumor antigens [29]. DC-CIK significantly enhanced the apoptosis ratio, depending on DC-CIK cell numbers, by increasing caspase-3 protein expression and reducing proliferating cell nuclear antigen protein expression against cancer stem cell [31]. These results support that a tumor marker can enhance the therapeutic effect of DC-CIK cells.

In vivo, PADI4-overexpressing ECA-109 cell lysate or rPADI4 cocultured CIK cells were injected into tumor-bearing mice can decline the tumor volume by 18.6% or 35.1% compared with the control group, which demonstrates that PADI4 can act as a tumor marker to enhance the therapeutic effect of DC-CIK cells in vivo. The significant therapeutic effect of DC-CIK cells on tumors is due to increased DC maturation, CIK cell proliferation, and cytotoxicity following stimulation with the PADI4 protein. Purification of the PADI4 protein can increase its ability to stimulate DC maturation and tumor growth suppression. These results also indicate that PADI4, not other proteins in ECA-109 cells, is responsible for the increased therapeutic efficiency in EC.

This study used TXNDC5 expression in ECA-109 cells as a nonspecific control. TXNDC5 shows increased expression in many tumors and plays an important role in tumorigenesis [32]. However, TXNDC5 overexpression did not significantly stimulate DC maturation, CIK cell development, or CIK cell cytotoxicity. These results demonstrate the specificity of PADI4 as a tumor marker to enhance the cytotoxicity of DC-CIK cells.

## 5. Conclusion

Experiments both in vitro and in an animal model demonstrate that incubation with PADI4 protein can significantly promote DC maturation, CIK cell proliferation, and elevate cytotoxicity and that PADI4-stimulated DC-CIK cells significantly suppressed tumor growth in tumor-bearing mice. These results suggest that the effect of PADI4 on tumor growth occurs via the promotion of DC maturation, CIK cell proliferation, and cytotoxicity. PADI4 may be an attractive target antigen for enhancing DC-based immunotherapy.

## Data Availability

The data used to support the findings of this study are available from the corresponding author upon request.

## Supplementary Materials

Supplementary 1 Supplementary Figure S1: detection of expression levels of PADI4 and TXNDC5 protein overexpressed in ECA-109 cells. (a) PADI4 or TXNDC5 cDNA was inserted into pcDNA3.1-RFP plasmids and expressed in ECA-109 cells. The PADI4, TXNDC5, and RFP expressions were detected using Western blot analysis. (b) The relative mRNA level of PADI4 was examined by real-time PCR. (c) The relative mRNA level of TXNDC5 was examined by real-time PCR. Asterisks indicate significant differences from the control group, and ^∗∗^ indicates *p* < 0.01.

Supplementary 2 Supplementary Figure S2: detection of CD3^+^CD4^+^, CD3^+^CD8^+^, and CD3^+^CD16^+^CD56^+^ CIK cells induced by RFP-, TXNDC5-, or PADI4-overexpressed ECA109 cell lysate-loaded DC using flow cytometry. CIK cells were induced with DCs loaded with lysate from RFP-overexpressing ECA-109 cells (OE-RFP) (a), TXNDC5-overexpressing ECA-109 cells (OE-TXNDC5) (b), or PADI4-overexpressing ECA-109 cells (OE-PADI4) (c). Statistical analysis of the above flow cytometry results (d).

Supplementary 3 Supplementary Figure S3: detection of recombinant expression of PADI4 in *E. coli* using SDS-PAGE. PADI4 gene was inserted into pGEx-4T1 plasmids and expressed in *E. coli* BL21. The PADI4 protein was expressed in a soluble form and purified using Glutathione Sepharose beads. The recombinant PADI4 protein was digested using protease K. The recombinant PADI4 protein (r-PADI4) and the digested recombinant protein (d-PADI4) were examined using SDS-PAGE with Coomassie brilliant blue staining.

Supplementary 4 Supplementary Figure S4: detection of CD3^+^CD4^+^, CD3^+^CD8^+^, and CD3^+^CD16^+^CD56^+^ CIK cells induced by d-rPADI4- or rPADI4-loaded DC using flow cytometry. (a) CIK cells were induced with DCs loaded with d-rPADI4. (b) CIK cells were induced with DCs loaded with rPADI4. (c) Statistical analysis of the above FCM results.
