# Supplementary figures and images for: Stimulation of DC-CIK with PADI4 Protein Can Significantly Elevate the Therapeutic Efficiency in Esophageal Cancer
Source: J Immunol Res. 2019 Mar 3;2019:6587570. doi: 10.1155/2019/6587570 (PMC6421725; doi:10.1155/2019/6587570)

## Slide 1
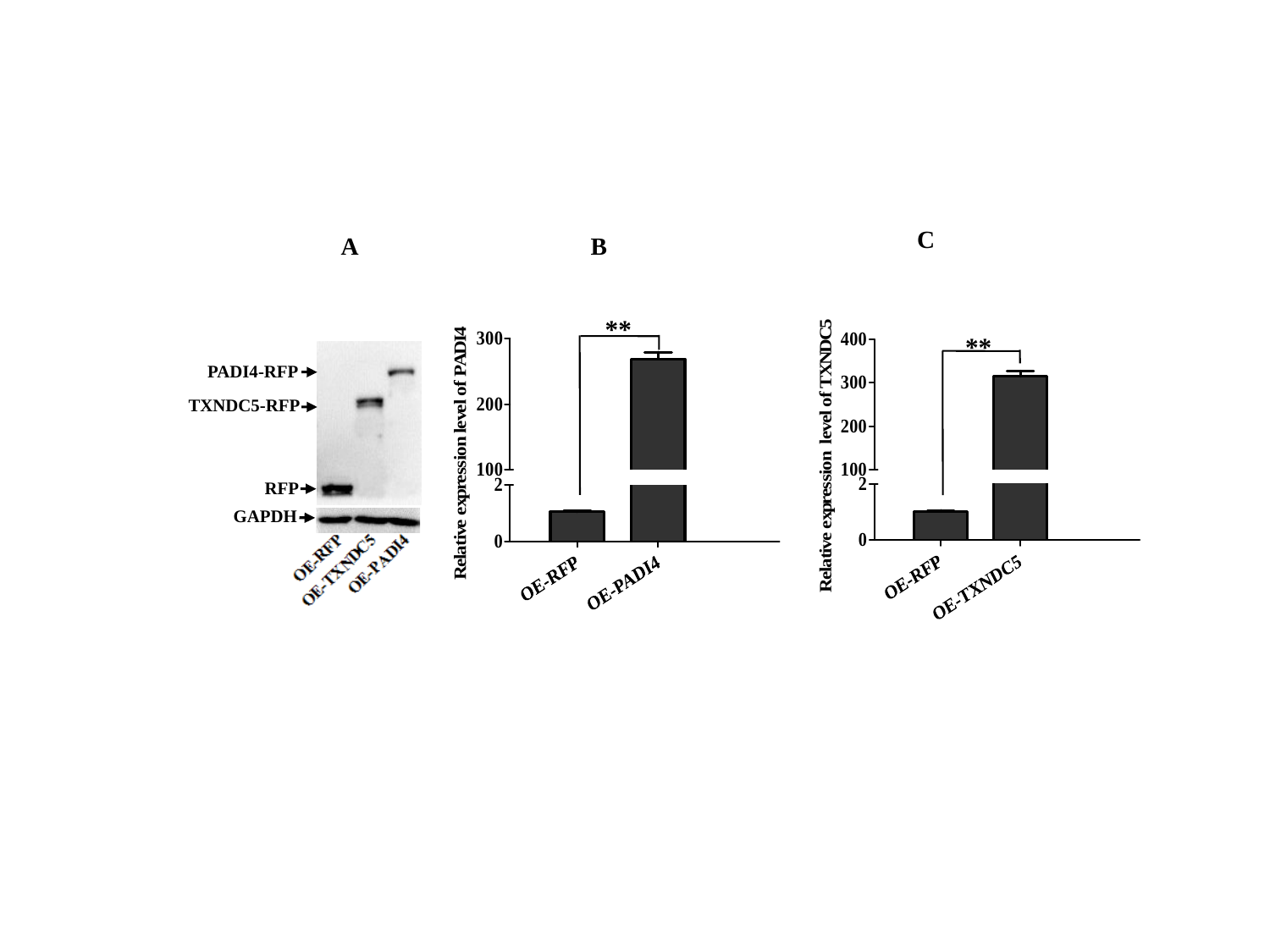

C
A
B
PADI4-RFP
TXNDC5-RFP
RFP
GAPDH
**
**

Supplement: Supplementary 1 — Supplementary Figure S1: detection of expression levels of PADI4 and TXNDC5 protein overexpressed in ECA-109 cells. (a) PADI4 or TXNDC5 cDNA was inserted into pcDNA3.1-RFP plasmids and expressed in ECA-109 cells. The PADI4, TXNDC5, and RFP expressions were detected using Western blot analysis. (b) The relative mRNA level of PADI4 was examined by real-time PCR. (c) The relative mRNA level of TXNDC5 was examined by real-time PCR. Asterisks indicate significant differences from the control group, and ∗∗ indicates p < 0.01. [file 6587570.f1.ppt]

## Slide 1
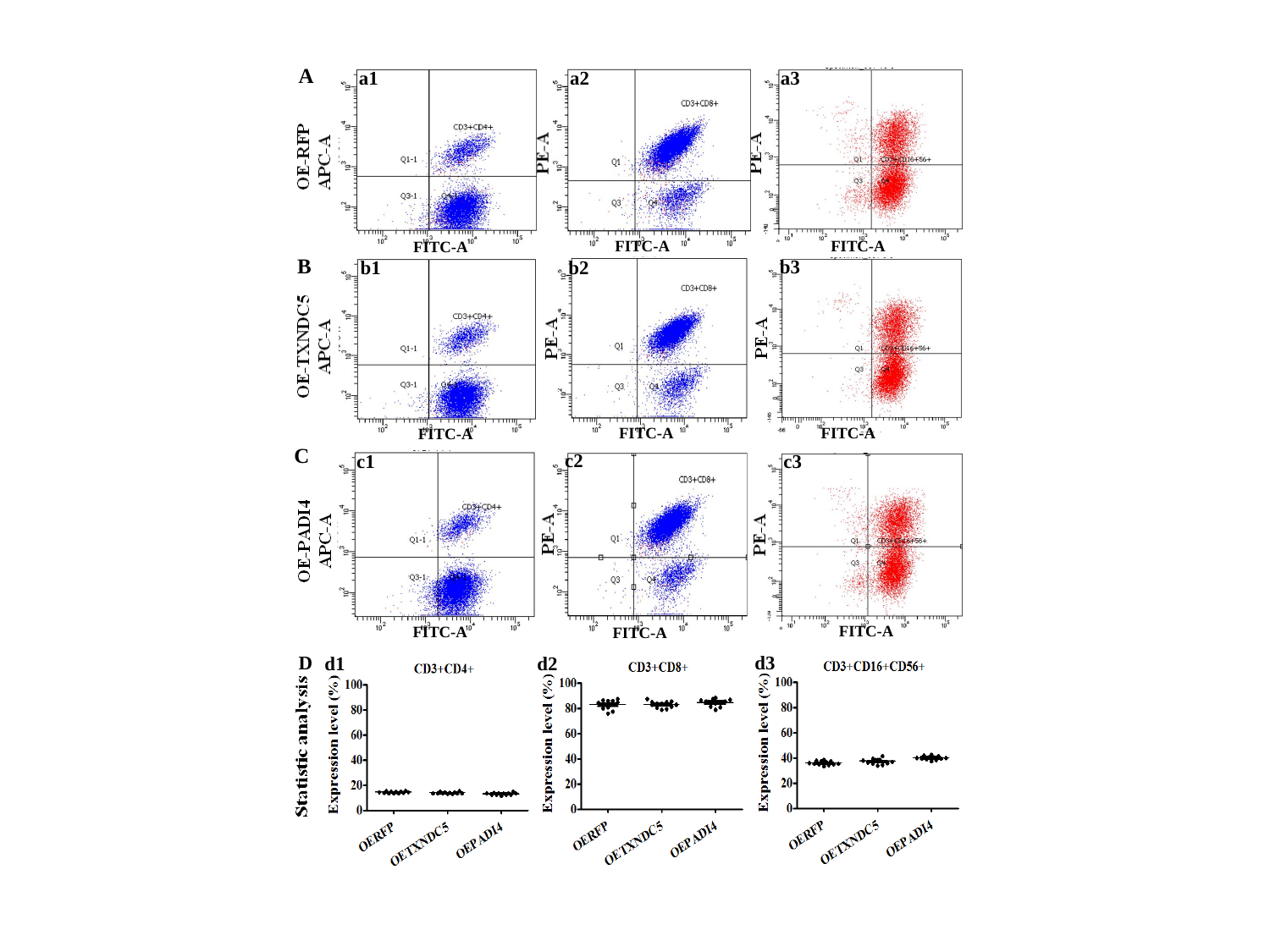

A
a1
a2
a3
FITC-A
FITC-A
FITC-A
B
b3
b1
b2
FITC-A
FITC-A
FITC-A
c2
FITC-A
c1
FITC-A
c3
FITC-A
D
d3
d2
d1
C

Supplement: Supplementary 2 — Supplementary Figure S2: detection of CD3+CD4+, CD3+CD8+, and CD3+CD16+CD56+ CIK cells induced by RFP-, TXNDC5-, or PADI4-overexpressed ECA109 cell lysate-loaded DC using flow cytometry. CIK cells were induced with DCs loaded with lysate from RFP-overexpressing ECA-109 cells (OE-RFP) (a), TXNDC5-overexpressing ECA-109 cells (OE-TXNDC5) (b), or PADI4-overexpressing ECA-109 cells (OE-PADI4) (c). Statistical analysis of the above flow cytometry results (d). [file 6587570.f2.ppt]

## Slide 1
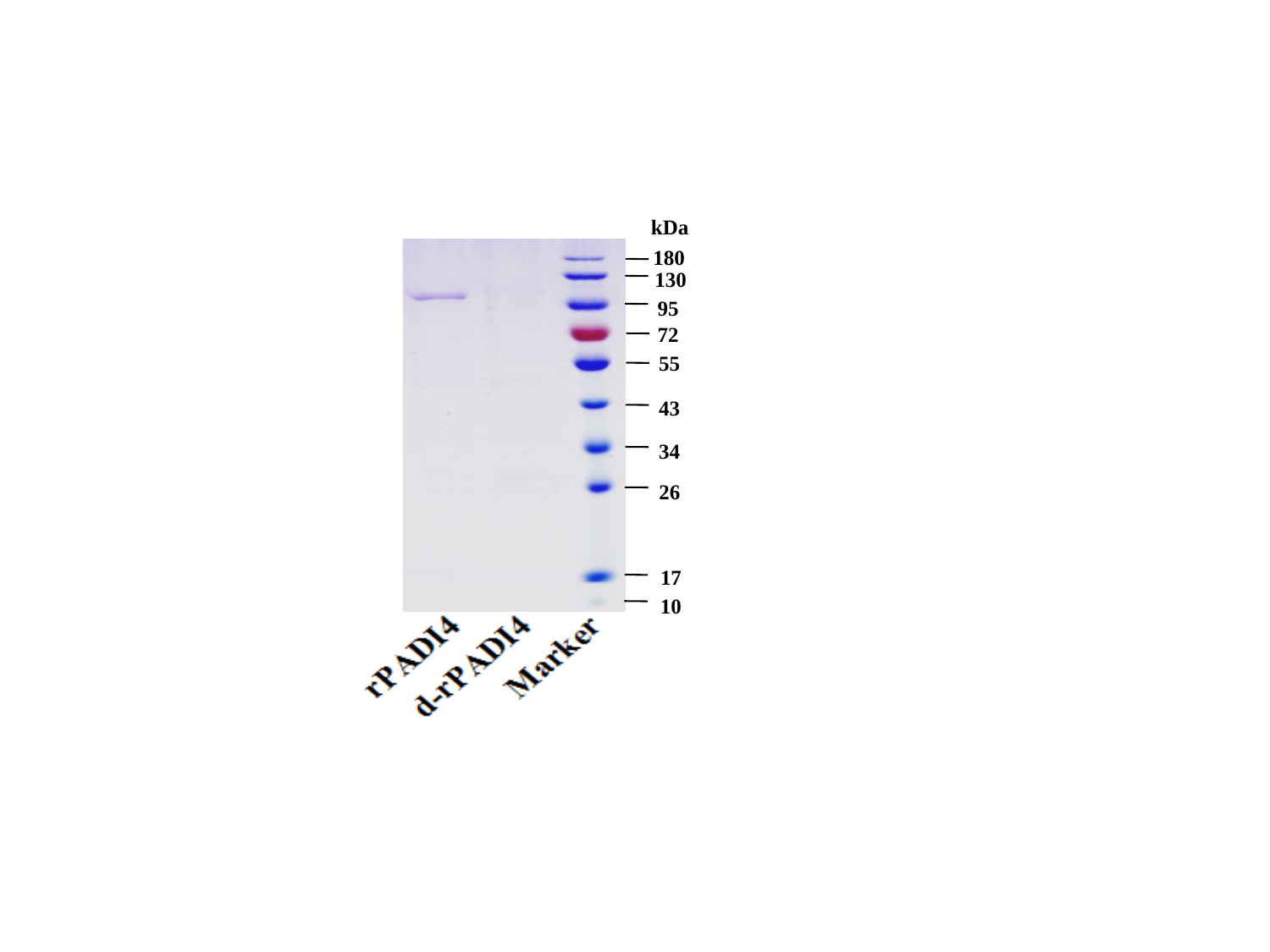

kDa
180
130
95
72
55
43
34
26
17
10

Supplement: Supplementary 3 — Supplementary Figure S3: detection of recombinant expression of PADI4 in E. coli using SDS-PAGE. PADI4 gene was inserted into pGEx-4T1 plasmids and expressed in E. coli BL21. The PADI4 protein was expressed in a soluble form and purified using Glutathione Sepharose beads. The recombinant PADI4 protein was digested using protease K. The recombinant PADI4 protein (r-PADI4) and the digested recombinant protein (d-PADI4) were examined using SDS-PAGE with Coomassie brilliant blue staining. [file 6587570.f3.ppt]

## Slide 1
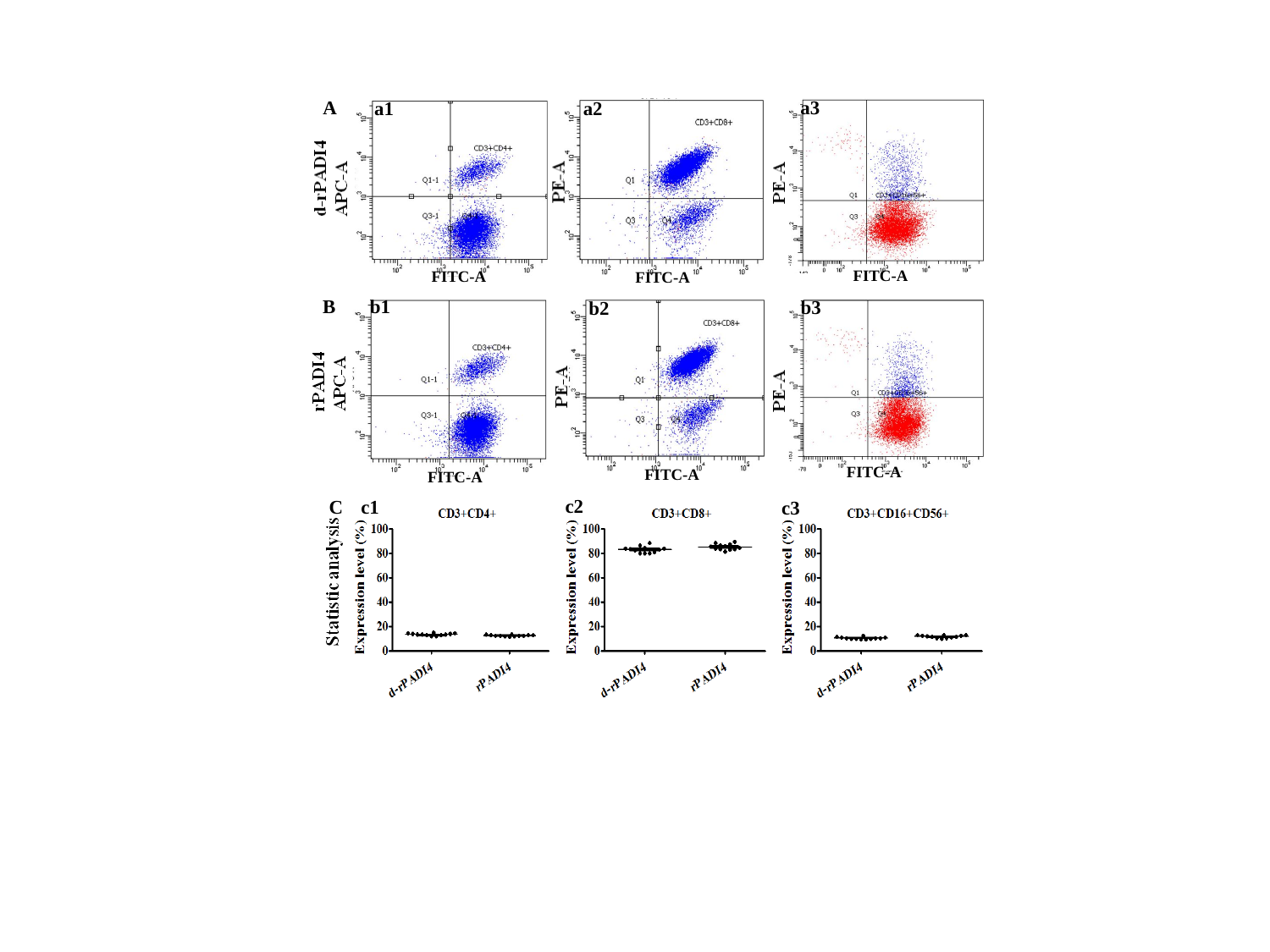

A
a1
FITC-A
a3
FITC-A
FITC-A
a2
B
FITC-A
b1
b3
FITC-A
b2
FITC-A
c2
c1
c3
C

Supplement: Supplementary 4 — Supplementary Figure S4: detection of CD3+CD4+, CD3+CD8+, and CD3+CD16+CD56+ CIK cells induced by d-rPADI4- or rPADI4-loaded DC using flow cytometry. (a) CIK cells were induced with DCs loaded with d-rPADI4. (b) CIK cells were induced with DCs loaded with rPADI4. (c) Statistical analysis of the above FCM results. [file 6587570.f4.ppt]
